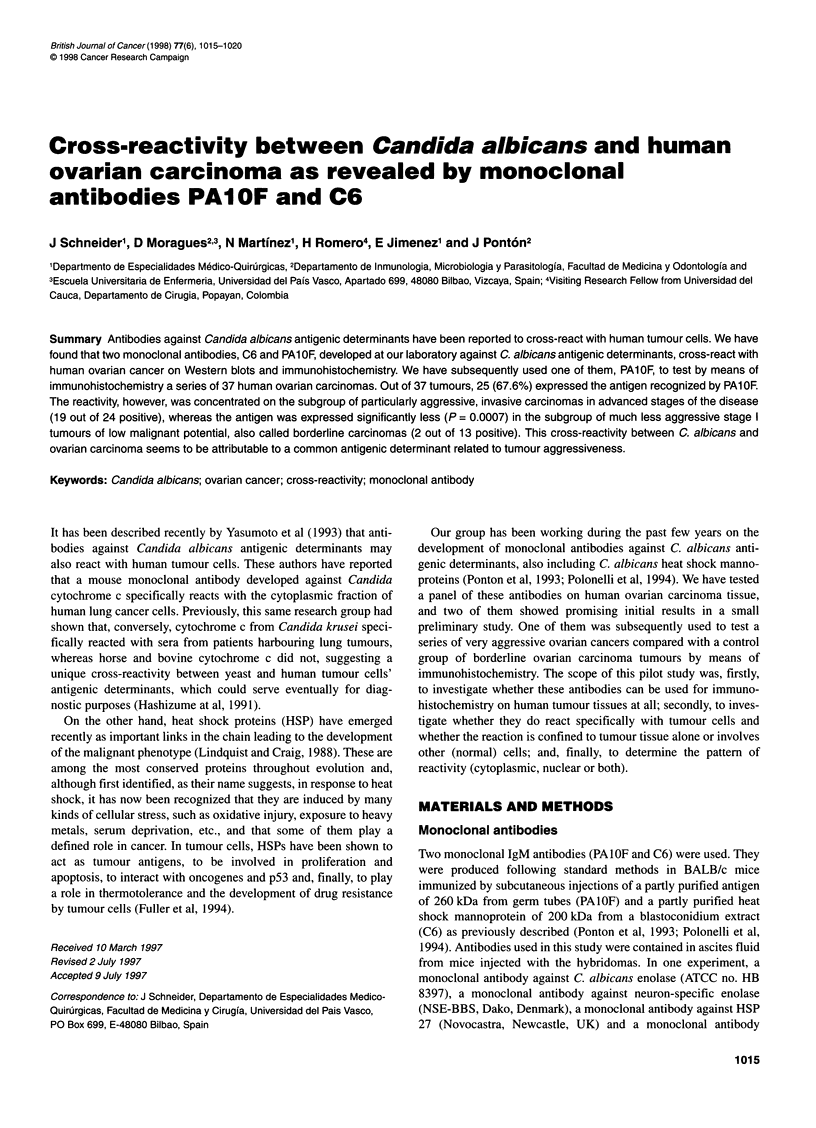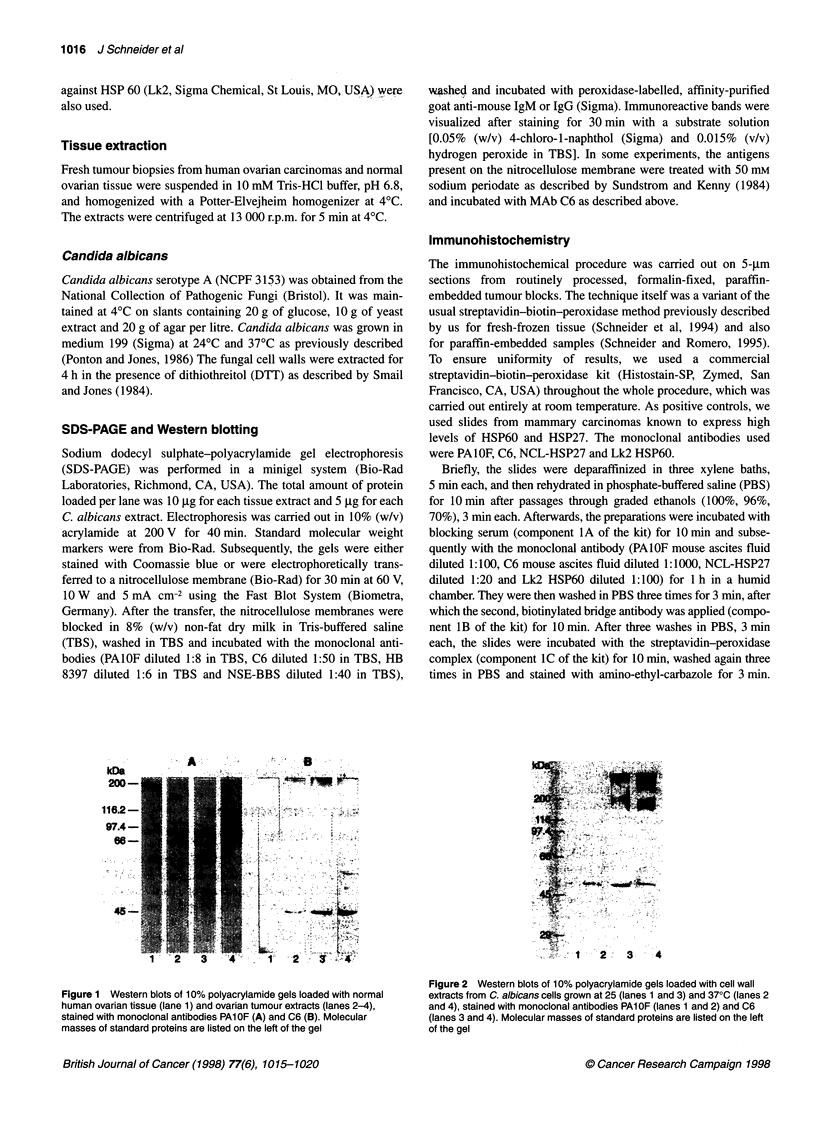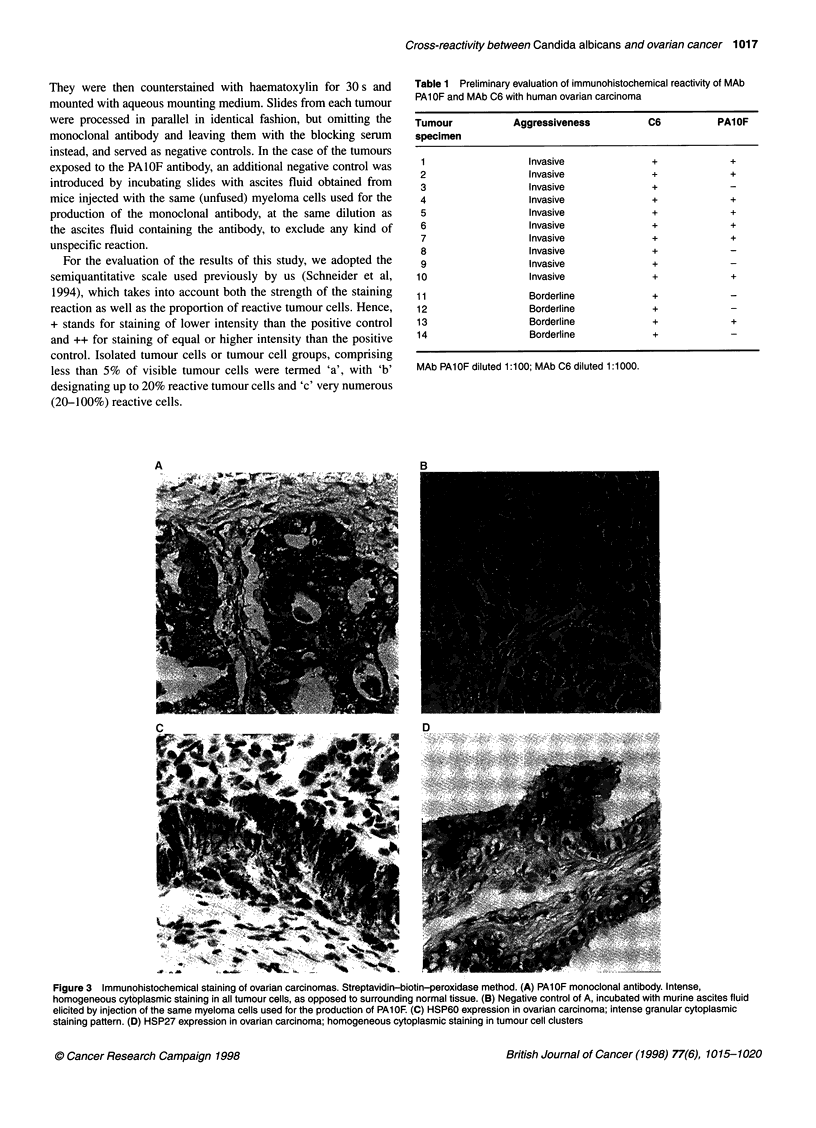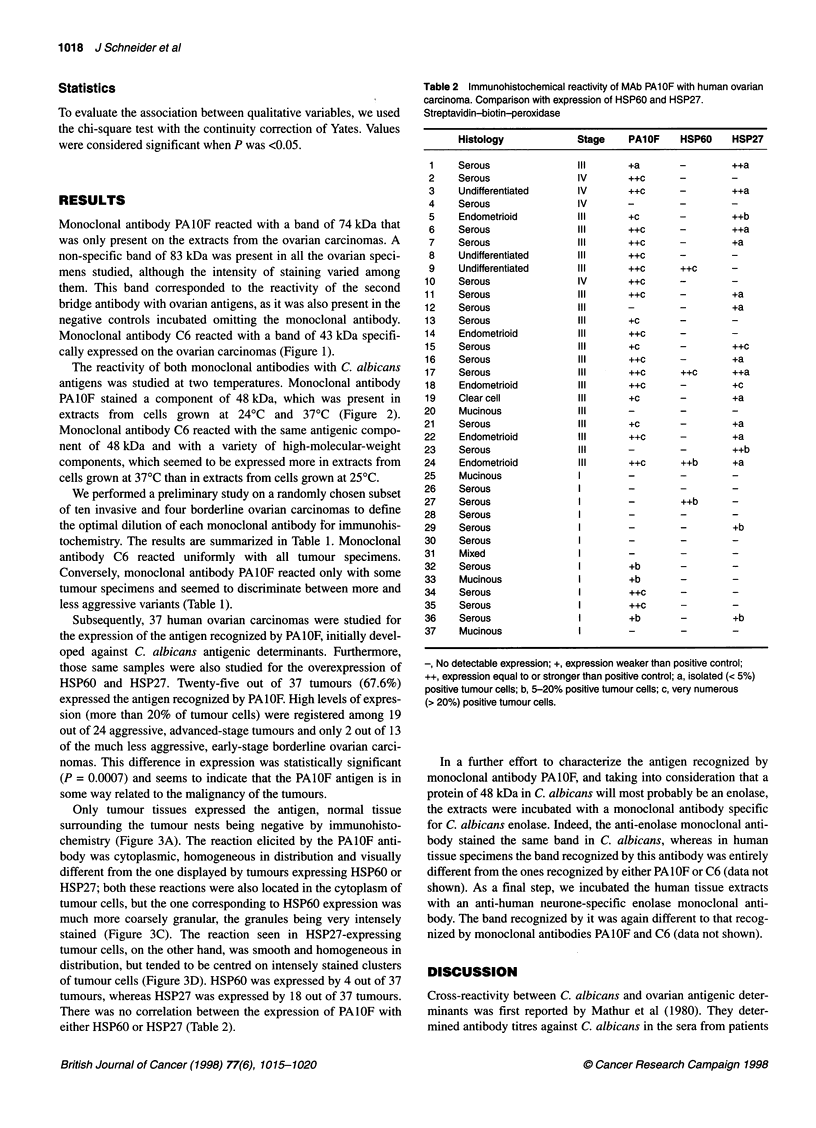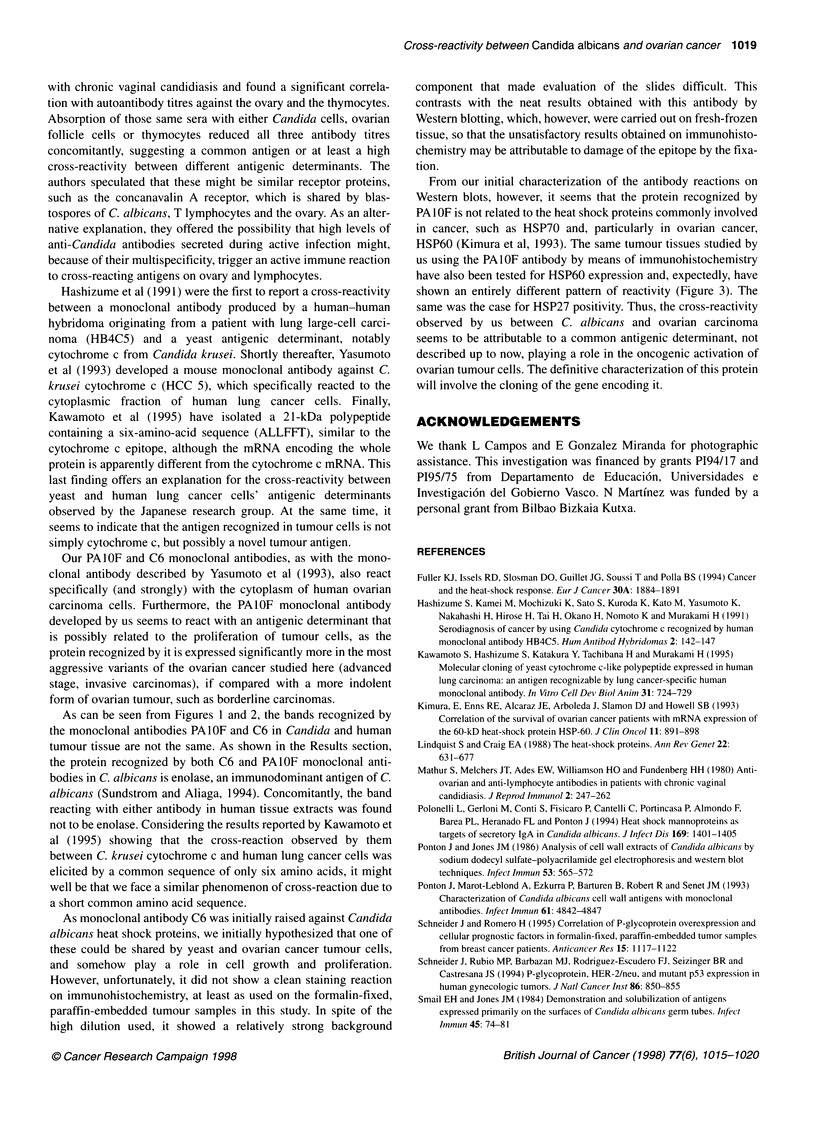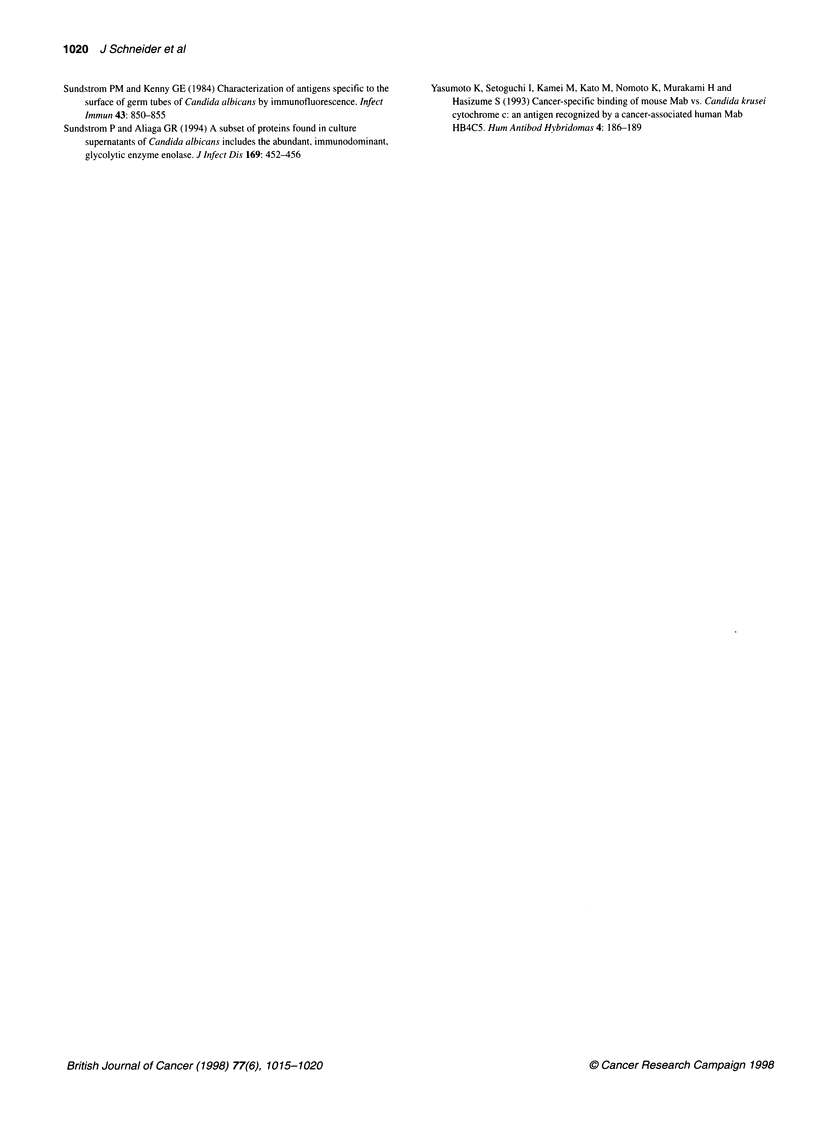# Cross-reactivity between Candida albicans and human ovarian carcinoma as revealed by monoclonal antibodies PA10F and C6.

**DOI:** 10.1038/bjc.1998.167

**Published:** 1998-03

**Authors:** J. Schneider, D. Moragues, N. MartÃ­nez, H. Romero, E. Jimenez, J. PontÃ³n

**Affiliations:** Departmento de Especialidades MÃ©dico-QuirÃºrgicas, Facultad de Medicina y OdontologÃ­a, Universidad del PaÃ­s Vasco, Bilbao, Vizcaya, Spain.

## Abstract

**Images:**


British Journal of Cancer (1998) 77(6), 1015–1020
© 1998 Cancer Research Campaign

### Cross-reactivity between *Candida albicans* and human ovarian carcinoma as revealed by monoclonal antibodies PA10F and C6

J Schneider[1], D Moragues[2,3], N Martínez[1], H Romero[4], E Jimenez[1] and J Pontón[2]

[1]Departmento de Especialidades Médico-Quirúrgicas, [2]Departamento de Inmunologia, Microbiologia y Parasitología, Facultad de Medicina y Odontología and [3]Escuela Universitaria de Enfermeria, Universidad del País Vasco, Apartado 699, 48080 Bilbao, Vizcaya, Spain; [4]Visiting Research Fellow from Universidad del Cauca, Departamento de Cirugia, Popayan, Colombia

**Summary** Antibodies against *Candida albicans* antigenic determinants have been reported to cross-react with human tumour cells. We have found that two monoclonal antibodies, C6 and PA10F, developed at our laboratory against *C. albicans* antigenic determinants, cross-react with human ovarian cancer on Western blots and immunohistochemistry. We have subsequently used one of them, PA10F, to test by means of immunohistochemistry a series of 37 human ovarian carcinomas. Out of 37 tumours, 25 (67.6%) expressed the antigen recognized by PA10F. The reactivity, however, was concentrated on the subgroup of particularly aggressive, invasive carcinomas in advanced stages of the disease (19 out of 24 positive), whereas the antigen was expressed significantly less ($P = 0.0007$) in the subgroup of much less aggressive stage I tumours of low malignant potential, also called borderline carcinomas (2 out of 13 positive). This cross-reactivity between *C. albicans* and ovarian carcinoma seems to be attributable to a common antigenic determinant related to tumour aggressiveness.

**Keywords:** *Candida albicans*; ovarian cancer; cross-reactivity; monoclonal antibody

It has been described recently by Yasumoto et al (1993) that antibodies against *Candida albicans* antigenic determinants may also react with human tumour cells. These authors have reported that a mouse monoclonal antibody developed against *Candida* cytochrome c specifically reacts with the cytoplasmic fraction of human lung cancer cells. Previously, this same research group had shown that, conversely, cytochrome c from *Candida krusei* specifically reacted with sera from patients harbouring lung tumours, whereas horse and bovine cytochrome c did not, suggesting a unique cross-reactivity between yeast and human tumour cells' antigenic determinants, which could serve eventually for diagnostic purposes (Hashizume at al, 1991).

On the other hand, heat shock proteins (HSP) have emerged recently as important links in the chain leading to the development of the malignant phenotype (Lindquist and Craig, 1988). These are among the most conserved proteins throughout evolution and, although first identified, as their name suggests, in response to heat shock, it has now been recognized that they are induced by many kinds of cellular stress, such as oxidative injury, exposure to heavy metals, serum deprivation, etc., and that some of them play a defined role in cancer. In tumour cells, HSPs have been shown to act as tumour antigens, to be involved in proliferation and apoptosis, to interact with oncogenes and p53 and, finally, to play a role in thermotolerance and the development of drug resistance by tumour cells (Fuller et al, 1994).

*Received 10 March 1997*
*Revised 2 July 1997*
*Accepted 9 July 1997*

*Correspondence to:* J Schneider, Departamento de Especialidades Medico-Quirúrgicas, Facultad de Medicina y Cirugía, Universidad del Pais Vasco, PO Box 699, E-48080 Bilbao, Spain

Our group has been working during the past few years on the development of monoclonal antibodies against *C. albicans* antigenic determinants, also including *C. albicans* heat shock mannoproteins (Ponton et al, 1993; Polonelli et al, 1994). We have tested a panel of these antibodies on human ovarian carcinoma tissue, and two of them showed promising initial results in a small preliminary study. One of them was subsequently used to test a series of very aggressive ovarian cancers compared with a control group of borderline ovarian carcinoma tumours by means of immunohistochemistry. The scope of this pilot study was, firstly, to investigate whether these antibodies can be used for immunohistochemistry on human tumour tissues at all; secondly, to investigate whether they do react specifically with tumour cells and whether the reaction is confined to tumour tissue alone or involves other (normal) cells; and, finally, to determine the pattern of reactivity (cytoplasmic, nuclear or both).

### MATERIALS AND METHODS

### Monoclonal antibodies

Two monoclonal IgM antibodies (PA10F and C6) were used. They were produced following standard methods in BALB/c mice immunized by subcutaneous injections of a partly purified antigen of 260 kDa from germ tubes (PA10F) and a partly purified heat shock mannoprotein of 200 kDa from a blastoconidium extract (C6) as previously described (Ponton et al, 1993; Polonelli et al, 1994). Antibodies used in this study were contained in ascites fluid from mice injected with the hybridomas. In one experiment, a monoclonal antibody against *C. albicans* enolase (ATCC no. HB 8397), a monoclonal antibody against neuron-specific enolase (NSE-BBS, Dako, Denmark), a monoclonal antibody against HSP 27 (Novocastra, Newcastle, UK) and a monoclonal antibody against HSP 60 (Lk2, Sigma Chemical, St Louis, MO, USA) were also used.

### Tissue extraction

Fresh tumour biopsies from human ovarian carcinomas and normal ovarian tissue were suspended in 10 mM Tris-HCl buffer, pH 6.8, and homogenized with a Potter-Elvejheim homogenizer at 4°C. The extracts were centrifuged at 13 000 r.p.m. for 5 min at 4°C.

### *Candida albicans*

*Candida albicans* serotype A (NCPF 3153) was obtained from the National Collection of Pathogenic Fungi (Bristol). It was maintained at 4°C on slants containing 20 g of glucose, 10 g of yeast extract and 20 g of agar per litre. *Candida albicans* was grown in medium 199 (Sigma) at 24°C and 37°C as previously described (Ponton and Jones, 1986) The fungal cell walls were extracted for 4 h in the presence of dithiothreitol (DTT) as described by Smail and Jones (1984).

### SDS-PAGE and Western blotting

Sodium dodecyl sulphate–polyacrylamide gel electrophoresis (SDS-PAGE) was performed in a minigel system (Bio-Rad Laboratories, Richmond, CA, USA). The total amount of protein loaded per lane was 10 µg for each tissue extract and 5 µg for each *C. albicans* extract. Electrophoresis was carried out in 10% (w/v) acrylamide at 200 V for 40 min. Standard molecular weight markers were from Bio-Rad. Subsequently, the gels were either stained with Coomassie blue or were electrophoretically transferred to a nitrocellulose membrane (Bio-Rad) for 30 min at 60 V, 10 W and 5 mA $cm^{-2}$ using the Fast Blot System (Biometra, Germany). After the transfer, the nitrocellulose membranes were blocked in 8% (w/v) non-fat dry milk in Tris-buffered saline (TBS), washed in TBS and incubated with the monoclonal antibodies (PA10F diluted 1:8 in TBS, C6 diluted 1:50 in TBS, HB 8397 diluted 1:6 in TBS and NSE-BBS diluted 1:40 in TBS), washed and incubated with peroxidase-labelled, affinity-purified goat anti-mouse IgM or IgG (Sigma). Immunoreactive bands were visualized after staining for 30 min with a substrate solution [0.05% (w/v) 4-chloro-1-naphthol (Sigma) and 0.015% (v/v) hydrogen peroxide in TBS]. In some experiments, the antigens present on the nitrocellulose membrane were treated with 50 mM sodium periodate as described by Sundstrom and Kenny (1984) and incubated with MAb C6 as described above.

### Immunohistochemistry

The immunohistochemical procedure was carried out on 5-µm sections from routinely processed, formalin-fixed, paraffin-embedded tumour blocks. The technique itself was a variant of the usual streptavidin–biotin–peroxidase method previously described by us for fresh-frozen tissue (Schneider et al, 1994) and also for paraffin-embedded samples (Schneider and Romero, 1995). To ensure uniformity of results, we used a commercial streptavidin–biotin–peroxidase kit (Histostain-SP, Zymed, San Francisco, CA, USA) throughout the whole procedure, which was carried out entirely at room temperature. As positive controls, we used slides from mammary carcinomas known to express high levels of HSP60 and HSP27. The monoclonal antibodies used were PA10F, C6, NCL-HSP27 and Lk2 HSP60.

Briefly, the slides were deparaffinized in three xylene baths, 5 min each, and then rehydrated in phosphate-buffered saline (PBS) for 10 min after passages through graded ethanols (100%, 96%, 70%), 3 min each. Afterwards, the preparations were incubated with blocking serum (component 1A of the kit) for 10 min and subsequently with the monoclonal antibody (PA10F mouse ascites fluid diluted 1:100, C6 mouse ascites fluid diluted 1:1000, NCL-HSP27 diluted 1:20 and Lk2 HSP60 diluted 1:100) for 1 h in a humid chamber. They were then washed in PBS three times for 3 min, after which the second, biotinylated bridge antibody was applied (component 1B of the kit) for 10 min. After three washes in PBS, 3 min each, the slides were incubated with the streptavidin–peroxidase complex (component 1C of the kit) for 10 min, washed again three times in PBS and stained with amino-ethyl-carbazole for 3 min.

**Figure 1** Western blots of 10% polyacrylamide gels loaded with normal human ovarian tissue (lane 1) and ovarian tumour extracts (lanes 2–4), stained with monoclonal antibodies PA10F (**A**) and C6 (**B**). Molecular masses of standard proteins are listed on the left of the gel

**Figure 2** Western blots of 10% polyacrylamide gels loaded with cell wall extracts from *C. albicans* cells grown at 25 (lanes 1 and 3) and 37°C (lanes 2 and 4), stained with monoclonal antibodies PA10F (lanes 1 and 2) and C6 (lanes 3 and 4). Molecular masses of standard proteins are listed on the left of the gel

© Cancer Research Campaign 1998

They were then counterstained with haematoxylin for 30 s and mounted with aqueous mounting medium. Slides from each tumour were processed in parallel in identical fashion, but omitting the monoclonal antibody and leaving them with the blocking serum instead, and served as negative controls. In the case of the tumours exposed to the PA10F antibody, an additional negative control was introduced by incubating slides with ascites fluid obtained from mice injected with the same (unfused) myeloma cells used for the production of the monoclonal antibody, at the same dilution as the ascites fluid containing the antibody, to exclude any kind of unspecific reaction.

For the evaluation of the results of this study, we adopted the semiquantitative scale used previously by us (Schneider et al, 1994), which takes into account both the strength of the staining reaction as well as the proportion of reactive tumour cells. Hence, + stands for staining of lower intensity than the positive control and ++ for staining of equal or higher intensity than the positive control. Isolated tumour cells or tumour cell groups, comprising less than 5% of visible tumour cells were termed 'a', with 'b' designating up to 20% reactive tumour cells and 'c' very numerous (20–100%) reactive cells.

**Table 1** Preliminary evaluation of immunohistochemical reactivity of MAb PA10F and MAb C6 with human ovarian carcinoma

| Tumour specimen | Aggressiveness | C6 | PA10F |
|---|---|---|---|
| 1 | Invasive | + | + |
| 2 | Invasive | + | + |
| 3 | Invasive | + | – |
| 4 | Invasive | + | + |
| 5 | Invasive | + | + |
| 6 | Invasive | + | + |
| 7 | Invasive | + | + |
| 8 | Invasive | + | – |
| 9 | Invasive | + | – |
| 10 | Invasive | + | + |
| 11 | Borderline | + | – |
| 12 | Borderline | + | – |
| 13 | Borderline | + | + |
| 14 | Borderline | + | – |

MAb PA10F diluted 1:100; MAb C6 diluted 1:1000.

**Figure 3** Immunohistochemical staining of ovarian carcinomas. Streptavidin–biotin–peroxidase method. (**A**) PA10F monoclonal antibody. Intense, homogeneous cytoplasmic staining in all tumour cells, as opposed to surrounding normal tissue. (**B**) Negative control of A, incubated with murine ascites fluid elicited by injection of the same myeloma cells used for the production of PA10F. (**C**) HSP60 expression in ovarian carcinoma; intense granular cytoplasmic staining pattern. (**D**) HSP27 expression in ovarian carcinoma; homogeneous cytoplasmic staining in tumour cell clusters

© Cancer Research Campaign 1998

### Statistics

To evaluate the association between qualitative variables, we used the chi-square test with the continuity correction of Yates. Values were considered significant when *P* was <0.05.

### RESULTS

Monoclonal antibody PA10F reacted with a band of 74 kDa that was only present on the extracts from the ovarian carcinomas. A non-specific band of 83 kDa was present in all the ovarian specimens studied, although the intensity of staining varied among them. This band corresponded to the reactivity of the second bridge antibody with ovarian antigens, as it was also present in the negative controls incubated omitting the monoclonal antibody. Monoclonal antibody C6 reacted with a band of 43 kDa specifically expressed on the ovarian carcinomas (Figure 1).

The reactivity of both monoclonal antibodies with *C. albicans* antigens was studied at two temperatures. Monoclonal antibody PA10F stained a component of 48 kDa, which was present in extracts from cells grown at 24°C and 37°C (Figure 2). Monoclonal antibody C6 reacted with the same antigenic component of 48 kDa and with a variety of high-molecular-weight components, which seemed to be expressed more in extracts from cells grown at 37°C than in extracts from cells grown at 25°C.

We performed a preliminary study on a randomly chosen subset of ten invasive and four borderline ovarian carcinomas to define the optimal dilution of each monoclonal antibody for immunohistochemistry. The results are summarized in Table 1. Monoclonal antibody C6 reacted uniformly with all tumour specimens. Conversely, monoclonal antibody PA10F reacted only with some tumour specimens and seemed to discriminate between more and less aggressive variants (Table 1).

Subsequently, 37 human ovarian carcinomas were studied for the expression of the antigen recognized by PA10F, initially developed against *C. albicans* antigenic determinants. Furthermore, those same samples were also studied for the overexpression of HSP60 and HSP27. Twenty-five out of 37 tumours (67.6%) expressed the antigen recognized by PA10F. High levels of expression (more than 20% of tumour cells) were registered among 19 out of 24 aggressive, advanced-stage tumours and only 2 out of 13 of the much less aggressive, early-stage borderline ovarian carcinomas. This difference in expression was statistically significant ($P$ = 0.0007) and seems to indicate that the PA10F antigen is in some way related to the malignancy of the tumours.

Only tumour tissues expressed the antigen, normal tissue surrounding the tumour nests being negative by immunohistochemistry (Figure 3A). The reaction elicited by the PA10F antibody was cytoplasmic, homogeneous in distribution and visually different from the one displayed by tumours expressing HSP60 or HSP27; both these reactions were also located in the cytoplasm of tumour cells, but the one corresponding to HSP60 expression was much more coarsely granular, the granules being very intensely stained (Figure 3C). The reaction seen in HSP27-expressing tumour cells, on the other hand, was smooth and homogeneous in distribution, but tended to be centred on intensely stained clusters of tumour cells (Figure 3D). HSP60 was expressed by 4 out of 37 tumours, whereas HSP27 was expressed by 18 out of 37 tumours. There was no correlation between the expression of PA10F with either HSP60 or HSP27 (Table 2).

**Table 2** Immunohistochemical reactivity of MAb PA10F with human ovarian carcinoma. Comparison with expression of HSP60 and HSP27. Streptavidin–biotin–peroxidase

| | Histology | Stage | PA10F | HSP60 | HSP27 |
|---|---|---|---|---|---|
| 1 | Serous | III | +a | – | ++a |
| 2 | Serous | IV | ++c | – | – |
| 3 | Undifferentiated | IV | ++c | – | ++a |
| 4 | Serous | IV | – | – | – |
| 5 | Endometrioid | III | +c | – | ++b |
| 6 | Serous | III | ++c | – | ++a |
| 7 | Serous | III | ++c | – | +a |
| 8 | Undifferentiated | III | ++c | – | – |
| 9 | Undifferentiated | III | ++c | ++c | – |
| 10 | Serous | IV | ++c | – | – |
| 11 | Serous | III | ++c | – | +a |
| 12 | Serous | III | – | – | +a |
| 13 | Serous | III | +c | – | – |
| 14 | Endometrioid | III | ++c | – | – |
| 15 | Serous | III | +c | – | ++c |
| 16 | Serous | III | ++c | – | +a |
| 17 | Serous | III | ++c | ++c | ++a |
| 18 | Endometrioid | III | ++c | – | +c |
| 19 | Clear cell | III | +c | – | +a |
| 20 | Mucinous | III | – | – | – |
| 21 | Serous | III | +c | – | +a |
| 22 | Endometrioid | III | ++c | – | +a |
| 23 | Serous | III | – | – | ++b |
| 24 | Endometrioid | III | ++c | ++b | +a |
| 25 | Mucinous | I | – | – | – |
| 26 | Serous | I | – | – | – |
| 27 | Serous | I | – | ++b | – |
| 28 | Serous | I | – | – | – |
| 29 | Serous | I | – | – | +b |
| 30 | Serous | I | – | – | – |
| 31 | Mixed | I | – | – | – |
| 32 | Serous | I | +b | – | – |
| 33 | Mucinous | I | +b | – | – |
| 34 | Serous | I | ++c | – | – |
| 35 | Serous | I | ++c | – | – |
| 36 | Serous | I | +b | – | +b |
| 37 | Mucinous | I | – | – | – |

–, No detectable expression; +, expression weaker than positive control; ++, expression equal to or stronger than positive control; a, isolated (< 5%) positive tumour cells; b, 5–20% positive tumour cells; c, very numerous (> 20%) positive tumour cells.

In a further effort to characterize the antigen recognized by monoclonal antibody PA10F, and taking into consideration that a protein of 48 kDa in *C. albicans* will most probably be an enolase, the extracts were incubated with a monoclonal antibody specific for *C. albicans* enolase. Indeed, the anti-enolase monoclonal antibody stained the same band in *C. albicans*, whereas in human tissue specimens the band recognized by this antibody was entirely different from the ones recognized by either PA10F or C6 (data not shown). As a final step, we incubated the human tissue extracts with an anti-human neurone-specific enolase monoclonal antibody. The band recognized by it was again different to that recognized by monoclonal antibodies PA10F and C6 (data not shown).

### DISCUSSION

Cross-reactivity between *C. albicans* and ovarian antigenic determinants was first reported by Mathur et al (1980). They determined antibody titres against *C. albicans* in the sera from patients

© Cancer Research Campaign 1998

with chronic vaginal candidiasis and found a significant correlation with autoantibody titres against the ovary and the thymocytes. Absorption of those same sera with either *Candida* cells, ovarian follicle cells or thymocytes reduced all three antibody titres concomitantly, suggesting a common antigen or at least a high cross-reactivity between different antigenic determinants. The authors speculated that these might be similar receptor proteins, such as the concanavalin A receptor, which is shared by blastospores of *C. albicans*, T lymphocytes and the ovary. As an alternative explanation, they offered the possibility that high levels of anti-*Candida* antibodies secreted during active infection might, because of their multispecificity, trigger an active immune reaction to cross-reacting antigens on ovary and lymphocytes.

Hashizume et al (1991) were the first to report a cross-reactivity between a monoclonal antibody produced by a human–human hybridoma originating from a patient with lung large-cell carcinoma (HB4C5) and a yeast antigenic determinant, notably cytochrome c from *Candida krusei*. Shortly thereafter, Yasumoto et al (1993) developed a mouse monoclonal antibody against *C. krusei* cytochrome c (HCC 5), which specifically reacted to the cytoplasmic fraction of human lung cancer cells. Finally, Kawamoto et al (1995) have isolated a 21-kDa polypeptide containing a six-amino-acid sequence (ALLFFT), similar to the cytochrome c epitope, although the mRNA encoding the whole protein is apparently different from the cytochrome c mRNA. This last finding offers an explanation for the cross-reactivity between yeast and human lung cancer cells' antigenic determinants observed by the Japanese research group. At the same time, it seems to indicate that the antigen recognized in tumour cells is not simply cytochrome c, but possibly a novel tumour antigen.

Our PA10F and C6 monoclonal antibodies, as with the monoclonal antibody described by Yasumoto et al (1993), also react specifically (and strongly) with the cytoplasm of human ovarian carcinoma cells. Furthermore, the PA10F monoclonal antibody developed by us seems to react with an antigenic determinant that is possibly related to the proliferation of tumour cells, as the protein recognized by it is expressed significantly more in the most aggressive variants of the ovarian cancer studied here (advanced stage, invasive carcinomas), if compared with a more indolent form of ovarian tumour, such as borderline carcinomas.

As can be seen from Figures 1 and 2, the bands recognized by the monoclonal antibodies PA10F and C6 in *Candida* and human tumour tissue are not the same. As shown in the Results section, the protein recognized by both C6 and PA10F monoclonal antibodies in *C. albicans* is enolase, an immunodominant antigen of *C. albicans* (Sundstrom and Aliaga, 1994). Concomitantly, the band reacting with either antibody in human tissue extracts was found not to be enolase. Considering the results reported by Kawamoto et al (1995) showing that the cross-reaction observed by them between *C. krusei* cytochrome c and human lung cancer cells was elicited by a common sequence of only six amino acids, it might well be that we face a similar phenomenon of cross-reaction due to a short common amino acid sequence.

As monoclonal antibody C6 was initially raised against *Candida albicans* heat shock proteins, we initially hypothesized that one of these could be shared by yeast and ovarian cancer tumour cells, and somehow play a role in cell growth and proliferation. However, unfortunately, it did not show a clean staining reaction on immunohistochemistry, at least as used on the formalin-fixed, paraffin-embedded tumour samples in this study. In spite of the high dilution used, it showed a relatively strong background component that made evaluation of the slides difficult. This contrasts with the neat results obtained with this antibody by Western blotting, which, however, were carried out on fresh-frozen tissue, so that the unsatisfactory results obtained on immunohistochemistry may be attributable to damage of the epitope by the fixation.

From our initial characterization of the antibody reactions on Western blots, however, it seems that the protein recognized by PA10F is not related to the heat shock proteins commonly involved in cancer, such as HSP70 and, particularly in ovarian cancer, HSP60 (Kimura et al, 1993). The same tumour tissues studied by us using the PA10F antibody by means of immunohistochemistry have also been tested for HSP60 expression and, expectedly, have shown an entirely different pattern of reactivity (Figure 3). The same was the case for HSP27 positivity. Thus, the cross-reactivity observed by us between *C. albicans* and ovarian carcinoma seems to be attributable to a common antigenic determinant, not described up to now, playing a role in the oncogenic activation of ovarian tumour cells. The definitive characterization of this protein will involve the cloning of the gene encoding it.

### ACKNOWLEDGEMENTS

We thank L Campos and E Gonzalez Miranda for photographic assistance. This investigation was financed by grants PI94/17 and PI95/75 from Departamento de Educación, Universidades e Investigación del Gobierno Vasco. N Martínez was funded by a personal grant from Bilbao Bizkaia Kutxa.

### REFERENCES

Fuller KJ, Issels RD, Slosman DO, Guillet JG, Soussi T and Polla BS (1994) Cancer and the heat-shock response. *Eur J Cancer* **30A**: 1884–1891

Hashizume S, Kamei M, Mochizuki K, Sato S, Kuroda K, Kato M, Yasumoto K, Nakahashi H, Hirose H, Tai H, Okano H, Nomoto K and Murakami H (1991) Serodiagnosis of cancer by using *Candida* cytochrome c recognized by human monoclonal antibody HB4C5. *Hum Antibod Hybridomas* **2**: 142–147

Kawamoto S, Hashizume S, Katakura Y, Tachibana H and Murakami H (1995) Molecular cloning of yeast cytochrome c-like polypeptide expressed in human lung carcinoma: an antigen recognizable by lung cancer-specific human monoclonal antibody. *In Vitro Cell Dev Biol Anim* **31**: 724–729

Kimura, E, Enns RE, Alcaraz JE, Arboleda J, Slamon DJ and Howell SB (1993) Correlation of the survival of ovarian cancer patients with mRNA expression of the 60-kD heat-shock protein HSP-60. *J Clin Oncol* **11**: 891–898

Lindquist S and Craig EA (1988) The heat-shock proteins. *Ann Rev Genet* **22**: 631–677

Mathur S, Melchers JT, Ades EW, Williamson HO and Fundenberg HH (1980) Anti-ovarian and anti-lymphocyte antibodies in patients with chronic vaginal candidiasis. *J Reprod Immunol* **2**: 247–262

Polonelli L, Gerloni M, Conti S, Fisicaro P, Cantelli C, Portincasa P, Almondo F, Barea PL, Heranado FL and Ponton J (1994) Heat shock mannoproteins as targets of secretory IgA in *Candida albicans*. *J Infect Dis* **169**: 1401–1405

Ponton J and Jones JM (1986) Analysis of cell wall extracts of *Candida albicans* by sodium dodecyl sulfate–polyacrilamide gel electrophoresis and western blot techniques. *Infect Immun* **53**: 565–572

Ponton J, Marot-Leblond A, Ezkurra P, Barturen B, Robert R and Senet JM (1993) Characterization of *Candida albicans* cell wall antigens with monoclonal antibodies. *Infect Immun* **61**: 4842–4847

Schneider J and Romero H (1995) Correlation of P-glycoprotein overexpression and cellular prognostic factors in formalin-fixed, paraffin-embedded tumor samples from breast cancer patients. *Anticancer Res* **15**: 1117–1122

Schneider J, Rubio MP, Barbazan MJ, Rodriguez-Escudero FJ, Seizinger BR and Castresana JS (1994) P-glycoprotein, HER-2/neu, and mutant p53 expression in human gynecologic tumors. *J Natl Cancer Inst* **86**: 850–855

Smail EH and Jones JM (1984) Demonstration and solubilization of antigens expressed primarily on the surfaces of *Candida albicans* germ tubes. *Infect Immun* **45**: 74–81

© Cancer Research Campaign 1998

Sundstrom PM and Kenny GE (1984) Characterization of antigens specific to the surface of germ tubes of *Candida albicans* by immunofluorescence. *Infect Immun* **43**: 850–855

Sundstrom P and Aliaga GR (1994) A subset of proteins found in culture supernatants of *Candida albicans* includes the abundant, immunodominant, glycolytic enzyme enolase. *J Infect Dis* **169**: 452–456

Yasumoto K, Setoguchi I, Kamei M, Kato M, Nomoto K, Murakami H and Hasizume S (1993) Cancer-specific binding of mouse Mab vs. *Candida krusei* cytochrome c: an antigen recognized by a cancer-associated human Mab HB4C5. *Hum Antibod Hybridomas* **4**: 186–189

*© Cancer Research Campaign 1998*